# *Hypericum foliosum* Quality Botanical and Chemical Markers and *In Vitro* Antioxidant and Anticancer Activities

**DOI:** 10.3390/plants12051087

**Published:** 2023-03-01

**Authors:** Gonçalo Infante Caldeira, Guanghong Zhang, Luís Pleno Gouveia, Mafalda Videira, Rita Serrano, Olga Silva

**Affiliations:** Research Institute for Medicines (iMed.ULisboa), Faculty of Pharmacy, Universidade de Lisboa, Av. Professor Gama Pinto, 1649-003 Lisboa, Portugal

**Keywords:** antioxidant, cytotoxicity, Hypericum foliosum, malfurada, medicinal plant, quality monograph, herbal medicine

## Abstract

*Hypericum foliosum* Aiton is an endemic Azorean *Hypericum* species. Even though the aerial parts of *Hypericum foliosum* are not described in any official pharmacopoeia, they are utilized in local traditional medicine due to their diuretic, hepatoprotective, and antihypertensive properties. This plant has previously been the subject of phytochemical characterization and has been studied for its antidepressant activity, showing significant results in animal models. The lack of a description of the main characteristics of the aerial parts, which would be necessary to properly identify this medicinal plant species, contributes to the possibility of misidentification events. We performed macroscopic and microscopic analyses that identified specific differential characteristics, such as the absence of dark glands, the dimensions of the secretory pockets in the leaf, and the presence of translucent glands in the powder. To continue our previous work on the biological activity of *Hypericum foliosum*, ethanol, dichloromethane/ethanol, and water extracts were prepared and studied for their antioxidant and cytotoxic activity. Extracts showed in vitro selective cytotoxic activity in human lung cancer cell line A549, colon cancer cell line HCT 8, and breast cancer cell line MDA-MB-231, with dichloromethane/ethanol showing higher activity against all cell lines (IC_50_ values of 71.49, 27.31, and 9.51 µg/mL, respectively). All extracts also showed significant antioxidant activity.

## 1. Introduction

*Hypericum foliosum* Aiton is a low shrub with large yellow flowers that belongs to the *Hypericaceae* family. This species is endemic to the Azores and belongs to the same section (*Androsaemum*) as *Hypericum androsaemum*, which is also used in Portuguese traditional medicine due to its diuretic, hepatoprotective, and antihypertensive properties [1]. Locally, *H. foliosum* is known as malfurada or furalha [2]. 

Currently, the *Hypericaceae* family comprises nine genera: *Cratoxylum* Blume, *Eliea* Cambess, *Harungana* Lamarck, *Hypericum* L., *Lianthus* N. Robson, *Santomasia* N. Robson, *Thornea* Breedlove & McClintock, *Triadenum* Rafinesque, and *Vismia* Vand. Most of the biodiversity within this family is included in the *Hypericum* genus (roughly 80%) [3,4]. However, this taxonomic classification has not been updated in the Flora Europea or in the Nova Flora de Portugal, in which the *Hypericum* genus is still part of the *Clusiaceae* (*Guttiferae*) [5].

The *Hypericum* genus comprises 484 species spread across all continents except for Antarctica. These species may exist as herbaceous or bushy species—or, rarely, as trees—and they are grouped into 36 taxonomic sections constructed according to specific combinations of morphological characteristics and biogeographic distribution. They are distributed across various habitats, from temperate regions to high mountains in the tropics, avoiding areas with extreme temperature, aridity, or salinity [6]. 

Many *Hypericum* species have long been used in traditional medicine and had their biological activities demonstrated through pharmacological studies. However, only the flowering part of *H. perforatum* is recognized as an herbal drug and described in the Portuguese and European Pharmacopoeias, and it was accepted as such by the European Medicines Agency (EMA) in 2009 [7,8]. In the last few years, a vast range of biological activities, such as anti-inflammatory, anticancer, antidiabetic, and antioxidant activities, related to plants from the *Hypericum* genus have been described in the published literature [9,10,11,12].

*H. foliosum* has long been a subject of study by our team. We previously conducted botanical, chemical, pharmacological, and toxicological studies using dried extracts of the flowering aerial parts of *H. foliosum***.** The results from our preliminary botanical analysis led to the description of the first anatomical features necessary for identifying this medicinal plant as an herbal drug. Other specimens harvested in different areas and seasons should also be observed to confirm the obtained data. In our chemical and pharmacological work on *H. foliosum*, we established the main secondary metabolites’ fingerprint profile with a methanolic extract. Phenolic compound derivatives, including biapigenin, catechin, chlorogenic acid, miquelianin, quercetin, and quinic acid, were identified. Antidepressant activity studies showed that the antidepressant activity of the *H. foliosum* methanolic extract was not inferior to those of *H. perforatum* and *H. androsaemum* in animal models. In addition, evaluation of chronic toxicity in vivo showed no significant impact on the liver, pancreas, kidneys, or lipid profile [1,7]. *H. foliosum* methanolic extract also showed strong radical scavenging and acetylcholinesterase-inhibitory activities [13,14].

Previous work by other authors on *H. foliosum* led to the identification of an acylphloroglucinol metabolite that demonstrated antimicrobial in vitro activity against *Staphylococcus aureus* [15]. The essential oil composition is known to mainly comprise *n-*nonane, limonene, terpinolene, and other terpene derivatives [2,7,13]. Conservation of the species was also a focus of study, with micropropagation methodologies showing good results [16].

To legitimize the use of *H. foliosum* as an herbal drug, it is essential to describe its botanical characteristics and establish a clear and universal quality control methodology through a quality herbal drug monograph, as well as deepen knowledge regarding its biological activity. Several secondary metabolites with diverse biological activities have previously been identified in *Hypericum* genus extracts. These compounds generally belong to classes such as acylphloroglucinols, flavonoids, phloroglucinols, and xanthones and exhibit in vitro anticancer, cell protection, anti-inflammatory, antimicrobial, and antidepressant activities [17]. Since the selective cytotoxicity and antioxidant activities related, respectively, to anticancer and cell protection mechanisms were among the most frequent biological activities described in studies focusing on this botanical genus, we decided to study these activities in *H. foliosum* ethanolic, dichloromethane:ethanol, and water extracts using in vitro models. We also aimed to continue our previous work and fully describe the distinctive macroscopic and microscopic characteristics of the raw material of *H. foliosum* aerial parts.

## 2. Results

### 2.1. Macroscopic Analysis

**Figure 1:**
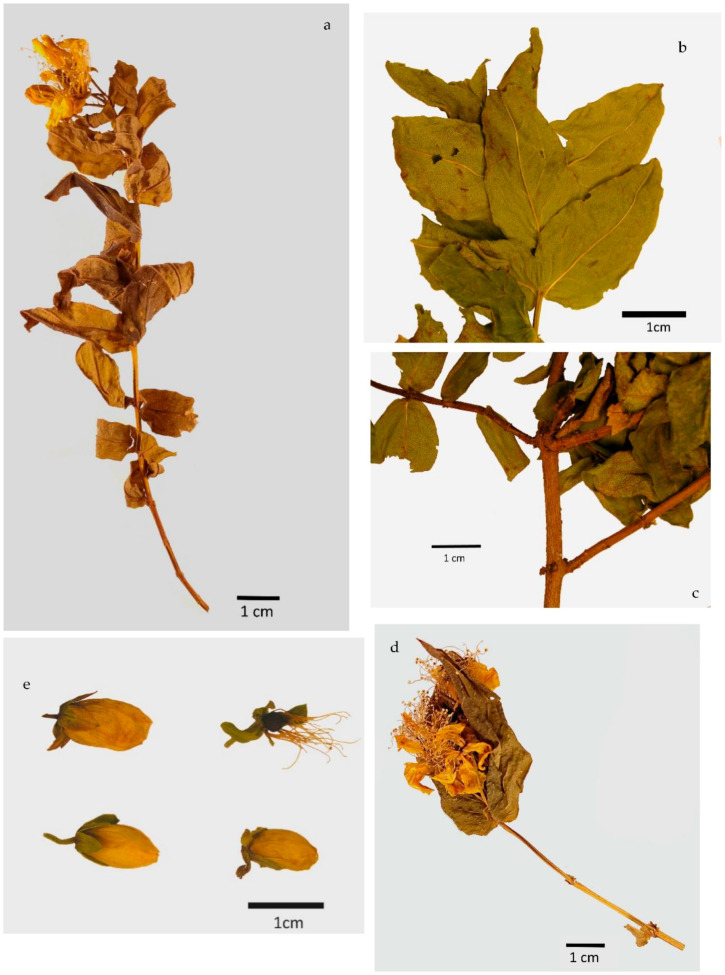
Macroscopic analysis photographs of the aerial parts of *H. foliosum*. 1 cm = 10 mm. (**a**) General view of the brown branched and bare stem and opposite and lanceolate leaves, with a whole margin and acute apex; (**b**) subsessile leaf details showing ovate-oblong form and stipules. The midrib originates at 45–60° angles from the secondary veins; (**c**) crenated stem surface with light brown color, dark pits, and longitudinal striation; (**d**) flowering aerial part; (**e**) different flowers showing yellow stamens surrounding three dark red carpels with lighter colored stigmas.

The macroscopic characterization of the aerial parts of *H. foliosum* (Table 1, Figure 1) revealed the presence of a yellowish-brown, branched, bare, and crenate stem (Figure 1a) with an average diameter of 3.5 ± 0.4 mm, prominent longitudinal ridges and dark pits scattered along the light brown surface (Figure 1b), and an internode distance of 2.0 ± 0.7 cm.

Opposite leaves were observed, showing ovate-oblong to lanceolate shapes, an entire margin, an acute apex, a length of 4.0 ± 0.6 mm and width 1.9 ± 0.4 mm, a short or almost inexistent petiole at the base, and yellowish-brown stipules (Figure 1c). The flowers were regular and the corymbs grouped (Figure 1d), with five green sepals with a brown base and lanceolate form, five orange-yellow petals, and long styles inserted at the base of the carpels (Figure 1e). Many pale-yellow stamens surrounded the three dark red carpels with lighter-colored stigmas (Figure 1e). Brown stilettos and ovaries were observed.

### 2.2. Microscopic Analysis

#### 2.2.1. Stem

**Figure 2:**
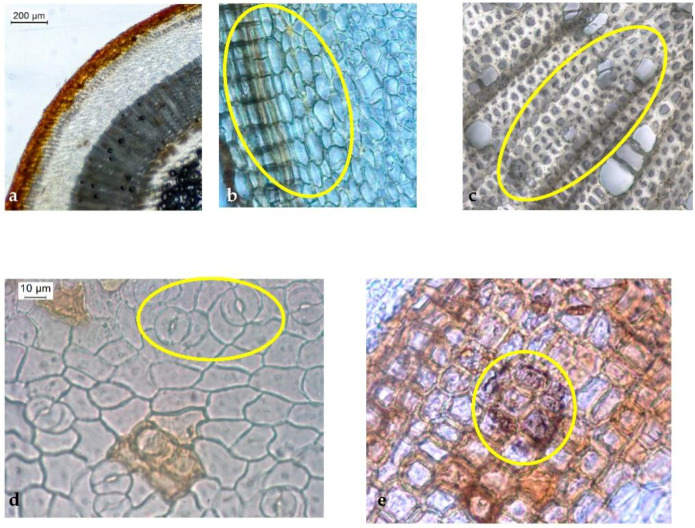
Light microscopy photographs of adult *H. foliosum* stem transverse section (**a**–**d**) and surface view (**e**). (**a**) General view showing four concentric rings with uneven distribution; (**b**) cortex region showing three to seven collenchymatous layers consisting of flattened cells and a type A secretory canal composed of four degenerated secretory cells and an evident lumen; (**c**) details of the xylem region associated with medullary rays; (**d**) epidermal strip detailing polyhedral phellem cells and paracytic stomata (oval selection); (**e**) details of brownish surface glands with a translucent interiors.

The main microscopic characteristics of the *H. foliosum* stem are presented in Table 2 and Figure 2. From the periphery to the interior, the transverse section of the *H. foliosum* adult stem (Figure 2a–e) showed four concentric rings with an uneven distribution. The outer ring consisted of a thick, red-brownish cuticle of phellem cells (cork cells) with striated walls (Figure 2a,b) followed by the external cortex, which had two to eight layers of laminar collenchyma consisting of rectangular and flattened cells. These layers were similar to the layer adjacent to the phellem, alternating with parenchyma cells of thinner walls (Figure 2c). In the inner cortex, there were a variety of roughly polyhedral parenchymatous cells. Dispersed by the cortical parenchyma, there were several types of secretory canals (or channels) composed of four degenerated secretory cells and an evident lumen with a diameter of 29.1 ± 8.7 μm (18.6 to 65.9 μm), forming a rosette-like structure (Figure 2c). More interiorly, vascular bundles radially occupying the major portion of the stem transverse section formed a shape like a circle. Phloem parenchymatous cells were neatly arranged in rows with a width of 48.0 ± 14.9 μm (17.1 to 95.4 μm). The secondary xylem was ring-porous, uniseriate, or multiseriate, occasionally containing distinctly heterogeneous xylem with large calibers, with an area of 639.4 ± 252.1 μm^2^ (210.5 to 1419.4 μm^2^). The parenchymatous medullary rays were depressed into the form of an elongated shape consisting of one to three rows of cells (Figure 2d). These pith rays were generally uniseriate, occasionally bi- or triseriate. In the inner ring, the pith was formed of round, thin-walled cells containing oval starch grains (Figure 2a). Compared to the adult stem, observation of the juvenile stem did not reveal significant morphological differences except for the presence of a lower degree of differentiation; a smaller lumen size and four companion cells, which constituted the initial formation of the narrow secretory canals, were observed. Observation of the epidermal strips of the *H. foliosum* stem (Table 2, Figure 2f) showed polyhedral and rectangular phellem cells from the surface view, with an average cell area of 639.8 ± 165.0 μm^2^. Specifically, a few paracytic stomata accompanied by two equal subsidiary cells and several surface glands showed brownish stains, and translucent interiors were also observed (Figure 2e). No calcium oxalate crystals were detected. With regard to the epidermal detachment of the stem and leaf, a significant difference was observed in the stem phellem cells, which were more rectangular and larger in size.

#### 2.2.2. Leaf

**Figure 3:**
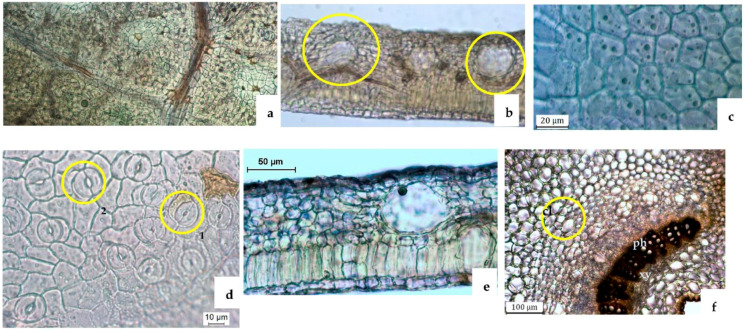
Light microscopy photographs of the *H. foliosum* leaf surface (**a**–**d**) and transverse section (**e**,**f**). (**a**) Adaxial surface view showing secondary veins forming a right angle with the primary veins, reticulated secondary venation, and translucent oil glands; (**b**) abaxial surface view showing translucent oil glands; (**c**) adaxial epidermal cells; (**d**) abaxial epidermal cells with corrugated walls and two types of stomata (1—anomocytic; 2—anisocytic); (**e**) leaf transverse section showing the bifacial mesophyll details; (**f**) transverse section of midrib showing circularly shaped collenchymatous cells and type B secretory canals, with a visible lumen scattered in the phloem, surrounded by four flattened cells (ph—phloem, xy—xylem).

The main microscopic characteristics of the *H. foliosum* leaf are presented in Table 3 and Figure 3. The top view of the *H. foliosum* leaf showed reticulated venation and translucent oil glands dispersed throughout the bifacial surface (Figure 3a,b). A midrib with a thickness of 719.3 ± 148.1 μm (294.0 to 882.1 μm) originated at angles of 45–60° from the secondary veins (Figure 3c). Secondary veins forming an angle of about 90° with the midrib (Figure 3a) were also observed. In Table 3, the main microscopic characteristics of the *H. foliosum* leaf are summarized. Microscopic analysis of the adaxial epidermis of the *H. foliosum* leaf clearly showed the presence of juxtaposed and polygonal cells, with an area of 317.4 ± 168.8 μm^2^ (79.3 to 802.3 μm^2^). No stomata were observed (Figure 3a,c). At the abaxial epidermis, a cell area of 382.4 ± 154.4 μm^2^ (187.1 to 650.9 μm^2^) was observed. When highlighted, this epidermis was revealed to be composed of cells with corrugated walls. Most of the observed stomata were anomocytic and anisocytic types and associated with two stomatal guard cells. Paracytic stomata were also found, but less frequently (Figure 3d). The abaxial stomatal index was 11.5 ± 3.6%. The microscopic analysis of the transverse section of the *H. foliosum* leaf (Figure 3e,f, Table 3) revealed several distinguishable characteristics; namely, a bifacial mesophyll (Figure 3e) formed by a single palisade layer of long cylindrical cells, sometimes regimented into two or three strata with similar lengths, and spongy parenchyma comprising several layers of branched cells that were not tightly connected. The mesophyll thickness was 126.9 ± 18.9 μm (94.3 to 177.9 μm), with a corresponding palisade parenchyma–spongy parenchyma ratio of 0.4 ± 0.1; the adaxial and abaxial epidermis consisted of one elongated layer of cells, covered by a smooth cuticle. The thickness of the adaxial cuticle was 4.0 ± 1.7 μm, similar to the observed abaxial cuticle value (3.6 ± 1.4 μm); several translucent glands with a diameter of 44.7 ± 11.2 μm (18.1 to 66.4 μm) were observed, mostly dispersed just between the abaxial epidermis and the spongy cells, showing a translucent oil content when observed from the top view (Figure 3a,b). The midrib had an elliptical shape with a thickness of 719.3 ± 148.1 μm (294.0 to 882.1 μm) and protruded from the underside. The abaxial view (Figure 3f,g) showed a large collenchyma area occupied by numerous circularly shaped cells with thick walls. The cambium was obviously distinguishable between the xylem and phloem, which contributed to a characteristic opened collateral vascular bundle. The thickness of the phloem was 63.8 ± 15.6 μm (40.5 to 92.5 μm). Some type B secretory canals were prominently scattered into the phloem, with a diameter of 32.2 ± 12.2 μm (11.2 to 59.7 μm) and a visible lumen surrounded by four flattened cells. The xylem, which had a thickness of 80.2 ± 8.8 μm (65.4 to 97.3 μm), was lying on the adaxial side of the vascular bundle and formed of lignified vessels of medium caliber. No calcium oxalate crystals were observed.

Despite the few paracytic stomata in the stem epidermis, the presence of stomata in the leaf, predominantly anomocytic and anisocytic, was crucial to distinguish between the adaxial and abaxial epidermis. However, there were no statistical differences between the adaxial and abaxial epidermis regarding single-cell dimensions. The presence of translucent oil glands dispersed throughout the leaf’s bifacial surface was easily observed in the powdered fragments, which was also an important characteristic for this medical raw-material identification.

#### 2.2.3. Powder

**Figure 4:**
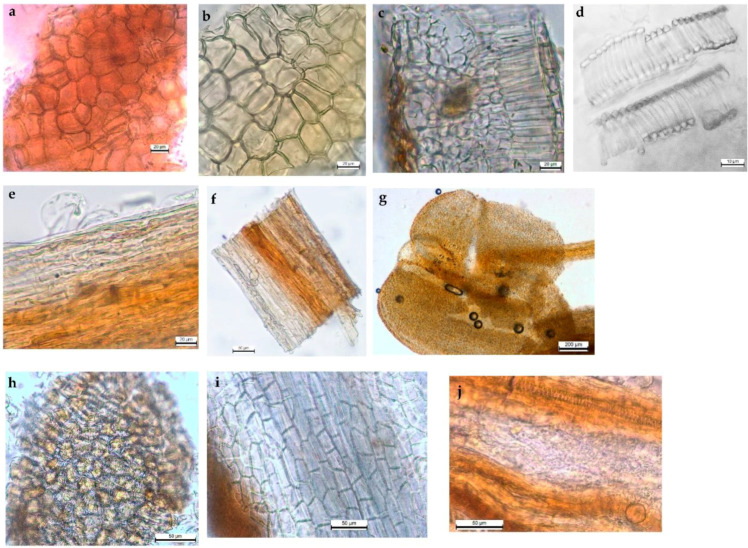
*H. foliosum* powder microscopic characteristics. (**a**) Stem phellem cells (top view); (**b**) stem parenchymatous cell (top view); (**c**) leaf bifacial mesophyll (transverse section); (**d**) tracheids and vessels (top view); (**e**) staminal filament (top view); (**f**) staminal filament (top view); (**g**) anthers (top view); (**h**) anthers (top view); (**i**) petal epidermis (top view); (**j**) stigma (transverse section).

The powdered drug produced from the *H. foliosum* aerial parts (Figure 4) presented a yellowish-green color and characteristic odor. Microscopically, it was identified by the presence of most of the characteristic elements of the leaf, stem, and flower; namely, fragments of the stem with yellowish-brown phellem cells; fragments of the central parenchyma of the stem with lignified and pitted rectangular cells; fragments of leaf bifacial mesophyll containing translucent glands inserted in palisade and spongy parenchyma; fragments of xylem with clusters of tracheids and punctuated vessels; fragments of the fibrous staminal filament with continuous thin-walled epidermal cells and striated cuticle fragments of anthers with countless yellow-brownish striated cuticle cells dotted in a stereo pod-shaped plane; fragments of obovate pollen grains with three germinal pores and a smooth exine, occurring singly or in dense groups; fragments of the petal epidermis with an elongated shape and straight or wavy anticlinal walls, distributed frequently with bright yellow oil glands, sometimes associated with small vessels; fragments of the black-red pistil strips covered by wrinkled and thick cuticles and with pollen tube-transmitting tracts in the center of the stigma (transverse section), associated with vessels; and fragments of the white ovary with a thick cuticle and filled with circular or rectangle parenchymatous cells. 

In the microscopic analysis of the powder, we not only completed the previous observations of the leaf and stem with the most frequent elements of the raw material but also found flower fragments with specific and identical characteristics, such as fragments of fibrous staminal filament, fragments of stereo pod-shaped anthers formed of striated cuticle cells, and fragments of the petal epidermis containing plenty of oil glands. We highlight the observation of fragments of pistils related to pollen tube-transmitting tracts in the center of the stigma (transverse section). On the other hand, our results revealed the presence of translucent glands, not only dispersed in the leaf’s spongy mesophyll but also in the palisade parenchyma. Furthermore, in the aerial parts of *H. foliosum*, pollen glands with three germinal pores and a smooth exine were identified, which were distinct from the *H. androsaemum* pollen grains (coarse exine) [18] but similar to those observed in the aerial parts of *H. perforatum* [19] (EDQM 2019). Additionally, when observed under a scanning electron microscope (SEM), the *H. foliosum* pollen grains were larger than those observed in *H. androsaemum* [18].

### 2.3. Antioxidant Activity

The results of the antioxidant activity evaluation of the aerial parts of *H. foliosum* using 2,2-diphenyl-1-picrylhydrazyl (DPPH), ferric reducing antioxidant power (FRAP), and phosphomolybdic acid (PA) colorimetric assays are presented in Table 4. Hydroethanolic (Hf.E), dichloromethane/methanol (Hf.DM), and water (Hf.W) extracts were used for analysis. 

All extracts showed antioxidant activity. However, the hydroethanolic extract (Hf.E) was the most active in terms of free radical scavenging capacity, and the dichloromethane extract (Hf.DM) was the extract with the higher total antioxidant capacity. A similar reducing power was observed with all the extracts. The Hf.W extract showed statistically (two-way ANOVA, alpha = 0.05) lower antioxidant activity than the Hf.E and DM extracts when assessed with the DPPH and PA methods. No discrimination between the extracts could be achieved when using the FRAP method (Table 4).

### 2.4. In Vitro Anticancer Activity

The results of the assessment of the anticancer activity of the aerial parts of *H. foliosum* against different cell lines are presented in Table 5.

Both Hf. E and Hf.DM showed selective anticancer activity against all human tumor cell lines tested, as seen in Table 5. Hf. W showed no effect under the tested conditions. The Hf.DM extract showed a statistically (two-way ANOVA, alpha = 0.05) lower cytotoxic activity than the Hf.E extract when assessed with any of the three methods used.

### 2.5. Chromatographic Profile

**Figure 5:**
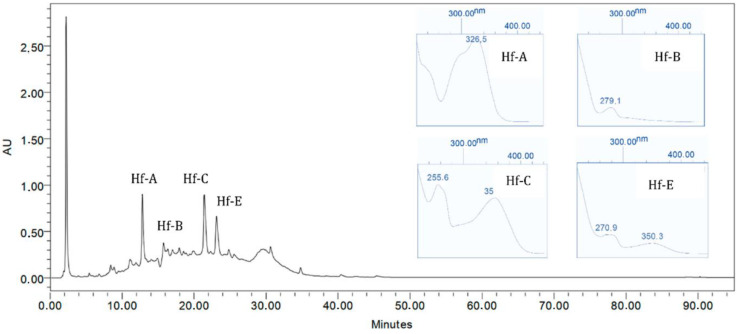
*Hypericum foliosum* hydroethanolic extract *maxplot* (λ = 190–450 nm) chromatogram.

**Figure 6:**
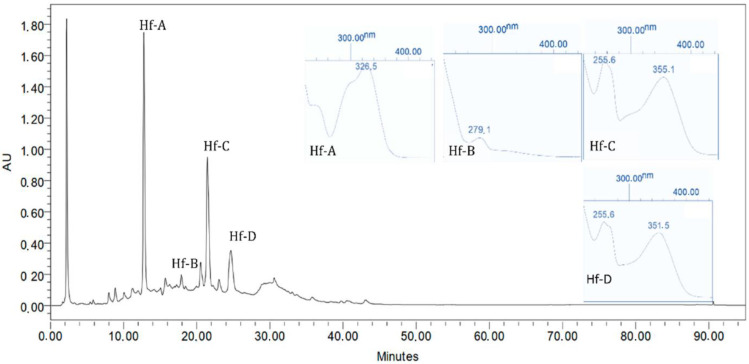
*Hypericum foliosum* DCM-MeOH extract *maxplot* (λ = 190–450 nm) chromatogram.

**Figure 7:**
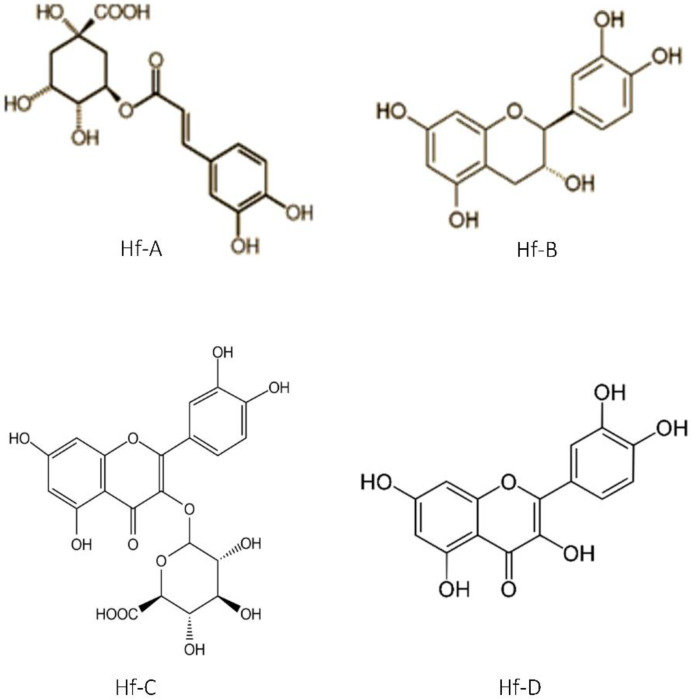
Compounds identified in *H. foliosum* DCM-MeOH extract; Hf-A chlorogenic acid; Hf-B catechin; Hf-C miquelianin; and Hf-D quercetin.

The obtained results can be seen in Figure 5 and Figure 6, where the UV spectra for the characteristic *Hypericum foliosum* compounds identified in the extracts under analysis are presented. A comparison with data generated in previous work by Machado with a different *H. foliosum* aerial part extract was undertaken [20]. Our analysis showed the presence of chlorogenic acid (Hf-A), catechin (Hf-B), miquelianin (Hf-C), and quercetin (Hf-D), which had already been isolated and identified in an *H. foliosum* aerial part extract. The chemical structures of these major compounds are presented in Figure 7. The presence of these marker compounds allowed for the confirmation of the phytochemical profile of *H. foliosum*. 

## 3. Discussion

In the present study, we describe the first complete and comprehensive macroscopic and microscopic characterization of herbal medicine produced from *H. foliosum* aerial part. 

Our macroscopic characterization of dried, fragmented raw material from *H. foliosum* aerial parts was consistent with the morphological botanical description of the species in the Nova Flora de Portugal by Franco et al. related to the species’ taxonomic identification [5]. The crenated stem with longitudinal ridges and dark pits; reticulated venation; stipules in the leaf’s basis; the number and color of sepals, petals, and carpels; and the absence of dark glands were the main features used for the species’ initial identification. 

Our microscopic analysis revealed distinct and useful characteristics for accurately identifying herbal medicine produced from *H. foliosum* aerial parts. For instance, in the transverse section view, we could see the stem with a thick cuticle; the number of collenchymatous layers, type A secretory canal, circularly shaped vascular bundles, and uniseriate medullary rays also contributed to a clear identification of *H. foliosum*. With regard to the leaf, the bifacial mesophyll with translucent glands, the open collateral vascular bundle, and the type B secretory canals in the protruded midrib were the most useful characteristics. As anticipated, our results shared several similarities with Serrano et al.’s findings [21]. 

To the best of our knowledge, this is the first full description of the stem, leaf, and flower of *H. foliosum*. No calcium oxalate crystals were observed. The outlines and proportions of vascular bundles in the stem and leaf are also important characteristics in distinguishing some *Hypericum* species. For instance, the xylem of the *Hipericum thymopsis* Boiss. fills the major part of the stem, whereas the pith is in the small region of the transverse section’s center [22], which can be observed easily. Our analysis of *H. foliosum*—specifically, the observation of circularly shaped vascular bundles in the stem and an open collateral vascular bundle type in the midrib of the leaf—can be used as important microscopic characteristics to differentiate *Hypericum* species and thus to identify *H. foliosum* aerial parts properly. 

A growing body of literature is focused on the secretory structure of *Hypericum* species [19,23,24,25,26]. Secretory canals seem to be a common secretory structure in the *Hypericum* sp. and play an important role in its anatomy and distinction. Generally, each species is characterized by the presence of different types of secretory structures, including translucent glands, black nodules, and secretory canals with different shapes, ontogenesis routes, and localizations [25]. In *H. foliosum*, the translucent glands were present within the lamina of the leaf, close to the abaxial surface; type A and type B secretory canals were found in the cortical parenchyma of the stem and associated with phloem in the midrib, respectively. These secretory structures and their localization have previously been described in other *Hypericum* sp., such as *Hypericum elodes* L. [20], *Hypericum perforatum* L. [16], *Hypericum inodorum* Mill., *Hypericum olympicum* L., and *Hypericum forrestii* (Chitt.) N. Robson, as well as in other families of Angiosperms [27]. 

In addition, even though the representative metabolic naphthodianthrones (such as hypericin) are only present in black nodules [22], histochemical tests have been used to establish a correlation between the function of secretory structures and the localization of some secondary metabolites [21]. From a pharmacological point of view, translucent glands and type B canals produce biologically secondary metabolites with potential pharmacological activity (such as alkaloids, lipids, and resins), which may protect the plant against herbivores and parasites. As tannins accumulate in stems [25], type A canals can act as storage cavities. 

Macroscopic analysis of new plant material harvested in 2021 confirmed the botanical characteristics previously observed, validating their use as monographic identification elements in a future quality monograph on *H. foliosum* aerial parts as an herbal drug.

Previous *H. foliosum* studies conducted by our team led to the identification of compounds such as quinic acid, catechin, chlorogenic acid, quercetin, miquelianin, and biapigenin in the methanolic extract and the establishment of a correlation between the antioxidant activity and flavonoid composition of the plant extract [20]. The higher the flavonoid content was, the stronger the antioxidant activity observed. Therefore, considering the antioxidant activity shown in our results, we can infer that the *H. foliosum* extracts under analysis in this study had different flavonoid contents, the ethanolic extract being the extract with the higher content, followed by dichloromethane/methanol 1:1. 

In this work, Hf. E and HF. DM showed significant anticancer activity against all cancer cell lines tested. Selectively, these extracts showed different levels of activity against MDA-MB-231, A549, and HCT8 cells, which are, respectively, related to breast, lung, and colon cancers. These results suggest a possible efflux of the main Hf. E secondary metabolites (phenol acid derivatives) by P-glycoprotein. 

The antioxidant and anticancer activities exhibited by *H. foliosum* in this study were in line with the major biological activities exhibited by natural compounds isolated from plants and subject to analysis, such as quercetin and catechin [17]. 

Our results are currently being investigated to achieve a better understanding of the cytotoxic activity exhibited by *H. foliosum* in this study. By establishing a correlation between class compounds and selective anticancer activities, we can contribute to the discovery of new compounds with potential therapeutic applications in oncology.

## 4. Material and Methods

### 4.1. Plant Material

Aerial parts of *H. foliosum* were collected during the flowering and fruiting season from the Flores and Corvo Islands, Azores, Portugal, between April and June 2016. The parts were collected in protected natural parks under the supervision of competent legal authorities since it is a protected species. The plant material was identified and dried by Eng. José Maria de Freitas from the Azorean Agricultural Development Services Island, currently named the Environment and Climate Change Service of S. Jorge. A voucher species was deposited in this institution. The plant material was stored in the Pharmacognosy Laboratory of the Faculty of Pharmacy, Universidade de Lisboa. All samples were dried at room temperature and protected from direct sunlight and moisture.

Forty samples of the aerial part of *H. foliosum* were randomly selected from 250 g of the collected raw material according to the standard methods of sampling described in the European Pharmacopoeia.

In 2021, we obtained new plant material harvested under the same conditions and performed the same macroscopic analyses.

### 4.2. Macroscopic Analysis (MA)

The 40 selected samples were examined macroscopically according to the standard methods described in the European Pharmacopoeia. The shape, size, color, and surface texture of the leaf, stem, and flower were the main characteristics observed. Samples were directly examined with the naked eye and then by using an Olympus SZ61 stereo microscope (Switzerland) coupled with a Leica MC170 HD digital camera controlled with the Leica Application Suite (LAS) Version 4.8.0 software (Switzerland). Images were acquired using this software.

### 4.3. Light Microscopy (LM)

Transverse sections of leaf lamina and midrib regions and epidermal strips of the leaves and stems from 20 representative samples of the selected plant material were prepared using conventional microscope techniques. Sections of the prepared samples were mounted in 60% aqueous chloral hydrate solution and examined using an Olympus CX31 microscope coupled with a Leica MC170 HD digital camera controlled with the LAS Version 4.8.0 software (Switzerland). The powdered plant material for the 20 samples was obtained using a water-cooled laboratory Analytical Mill A-10 (IKA) and observed using the same equipment under the same conditions as for the sample sections. Images were also acquired using this software and edited using Lightroom software.

### 4.4. Quantitative and Statistical Analysis

Quantification of selected morphological and anatomical characteristics was performed using LAS software (Switzerland). Statistical values were calculated using Microsoft Excel 2016 software. All macro- and microscopic results were expressed as means ± SD (Min (minimum) to Max (maximum)), except for the determination of the spongy parenchyma–palisade parenchyma ratio and the stomatal index. The stomatal index (SI) was determined using the formula SI = (S × 100)/(S + E), where (S) represents the number of stomata in a given area of a leaf and (E) the number of epidermal cells (including trichomes) in the same area of the leaf [19] (EDQM, 2019).

### 4.5. Extract Preparation

Aerial parts of *H. foliosum* were ground into a coarse powder in accordance with the European Pharmacopoeia 9 (2016). The powdered drug used in the botanical identification protocol was obtained via pulverization in a porcelain mortar using a pestle until a degree of fineness equating to coarse powder was reached. About 324 g of powder was obtained and uniformly divided into two portions of 162 g:

- The first portion was used for the whole-extract preparation (**Hf. E**), adding ten times the amount of cold ethanol (70%) and soaking it for 24 h. This procedure was repeated until the total raw material was exhausted. The obtained solution was filtered and evaporated under reduced pressure (T < 40 °C) in a rotary evaporator. The **Hf. E** total extract was kept in a desiccator at room temperature and protected from light;
- The second portion was used for the dichloromethane/methanol 1:1 (**Hf. DM**) and water (**Hf. W**) extracts. The raw material was completely covered with 1:1 DCM/methanol for 24 h. The **Hf. DM** extract was obtained after evaporating both solutions under pressure with a rotary vacuum flask evaporator;
- After the extraction with DCM/methanol and methanol, the final obtained residue was re-extracted with ultrapure water for 24 h. The obtained solution was freeze dried for 2~3 days and lyophilized (**Hf. W**).

### 4.6. Biochemical Antioxidant Assays

#### 4.6.1. DPPH (2,2-Diphenyl-1-picrylhydrazyl) Assay for Free Radical Scavenging Activity

This test was performed according to the method described by Blois (1958) with the slight modifications suggested by Brand-Williams et al. (1995). Diluted extracts (100 µL) of *H. foliosum* (**Hf. E**, **Hf. DM**, **Hf. W**) in different concentrations were added to 3.9 mL of DPPH solution (6 × 10^–5^ M in methanol). After 30 min of incubation in the dark at room temperature, the absorbance was measured at 517 nm. The reference standard was ascorbic acid. The free radical scavenging activity (% antiradical activity) was calculated using the following equation: % antiradical activity = (Acontrol − Asample)/Acontrol × 100, where Acontrol was the absorbance of the control test (containing all reagents except the sample) and Asample was the absorbance of the extract. Each experiment was carried out in triplicate, and the results were expressed as the mean percentage antiradical activity ± SD (n = 3) and presented as the inhibitory concentration (IC_50_ value), which represents the concentration of the sample required to scavenge 50% of the DPPH free radicals.

#### 4.6.2. Ferric Reducing Antioxidant Power (FRAP) Assay for Reducing Power

For the ferric reducing antioxidant power (FRAP) assay, diluted extracts of *H. foliosum* (**Hf. E**, **Hf. DM**, and **Hf. W**) (100 µL) in different concentrations were mixed with 3 mL of freshly prepared FRAP reagent (0.3 M acetate buffer (pH = 3.6), 10 mM 2,4,6-tripyridyl-s-triazine (TPTZ) in 40 mM HCl, and 20 mM FeCl_3_·6H_2_O were mixed in a ratio of 10:1:1 (*v*/*v*/*v*) and warmed to 37 °C before use). After 4 min of incubation at 37 °C, the absorbance was measured at 593 nm. Ascorbic acid was used as a standard. The results were expressed as ascorbic acid equivalents (mg AAE/g dE) and calculated as the mean value ± SD (n = 3).

#### 4.6.3. Phosphomolybdic Acid (PA) Assay for Total Antioxidant Activity

Diluted extracts of *H. foliosum* (**Hf. E**, **Hf. DM**, and **Hf. W**) (300 µL) in different concentrations were mixed with 3 mL of reagent solution containing 0.6 M sulfuric acid, 28 mM sodium phosphate, and 4 mM ammonium molybdate. After 90 min of incubation at 95 °C, the absorbance was measured at 695 nm with a Hitachi U-2000 UV–Vis spectrophotometer (Tokyo, Japan). Ascorbic acid was used as a standard, and the results were expressed as the inhibitory concentration (IC_50_ value).

### 4.7. Anticancer Activity

#### 4.7.1. Tumor Cell Lines

Anticancer activity tests were performed using three cancer cell lines: the human lung cancer cell line A549, the colon cancer cell line HCT 8, and the breast cancer cell line MDA-MB-436. All were obtained from the American Type Culture Collection (Manassas, VA, USA) and maintained under appropriate conditions.

#### 4.7.2. MTT Assay

For the cell viability assays, 10,000 cells/well were seeded in a 96-well plate. After 24 h, the medium was removed and washed with 100 μL of 1× PBS (VWR; Portugal). Cells were then incubated without (negative control) or with the extracts (**Hf. E**, **Hf. DM**, and **Hf. W**) in concentrations ranging from 25 to 200 µg/mL for 24 h. At the end of this time, the medium was removed and each well was washed with 100 μL of 1× PBS. Cell viability was characterized using the MTT assay. Briefly, cells were incubated for 3 h with 200 μL of MTT (0.5 mg/mL non-supplemented medium/well) (VWR; Portugal) and then lysed with 100 μL of DMSO (VWR; Portugal). Absorbance (570 nm) was measured with a FLUOstar^®^ Omega MicroPlate Reader. Cell viability was determined using the formula: cell viability % = [−(OD(experimental) −OD(blank))/(OD(control) − OD(blank)) × 100] (OD: optical density). Cytotoxicity was expressed as the concentration of the extract inhibiting cell growth by 50% (IC_50_). All experiments were undertaken in triplicate.

### 4.8. LC-UV/DAD Chromatographic Profile

To conduct our experimental work, samples of the extracts were reconstituted in an acetonitrile/water mixture to a concentration of 18.2 mg/mL. After that, samples were filtered with a 0.22 μm filter and submitted to chromatographic analysis at 28 °C using a Waters Symmetry (3.9 × 150 mm, 5 μm) protected with a Waters Symmetry pre-column (3.9 × 20 mm, 5 μm). The gradient elution was performed with a 0.8 mL/min flow rate using solvent A (0.05% *v*/*v* trifluoroacetic acid (TFA) in water (H_2_O)), solvent B (acetonitrile (MeCN)), and solvent C (MeOH) and the gradient program presented in Table 6. Peaks were detected at the maximum intensity and *maxplot* chromatograms were generated using Waters Millenium 32 software. HPLC-UV/DAD analysis was performed using a Waters Alliance 2690 Separations Module (Waters Corporation, Milford, MA, USA) coupled with a Waters 996 photodiode array detector (UV/DAD) (Waters Corporation, MA).

## 5. Conclusions

Our botanical characterization provides valuable information to be included in identification protocols for *H. foliosum* as a raw material for industrial use and offers additional support for further biochemical studies concerning this medicinal plant. Since the botanical specificities of *H. foliosum* are now well-described, quality control of the plant can be standardized. Our results regarding the antioxidant and selective anticancer activities of *H. foliosum* extracts are currently the subject of research intended to provide a better characterization of the class compounds responsible for the exhibited biological activities.

## Figures and Tables

**Table 1:** *H. foliosum* leaf and stem dimensions.

Statistical Parameters, n = 40		Min	Max	Mean	Median	±SD
**Leaf Size**	Length (cm)	2.8	5.3	4.0	4.0	0.6
	Width (cm)	1.1	2.8	1.9	1.9	0.4
**Stem Size**	Diameter (mm)	2.6	4.3	3.5	3.4	0.4
	Internode distance (cm)	0.8	4.0	2.0	1.8	0.7

Min, minimum; Max, maximum; N, number of samples; SD, standard deviation.

**Table 2:** Light microscopic characteristics of *H. foliosum* stem.

Statistical Parameters N = 20	Min	Max	Mean	Median	±SD
**Stem Surface**					
Epidermal cell area (µm^2^)	254.7	1053.1	639.8	653.8	165.0
**Transverse Section**					
Phloem thickness (µm)	17.1	95.4	48.0	47.9	14.9
Xylem vessel area (µm^2^)	210.5	1419.4	639.4	627.1	252.1
Epidermal cell layers (number)	2.0	8.0	4.4	4.0	1.4
Medullary ray width (cell number)	1.0	3.0	1.2	1.0	0.5
Secretory canals—type A (diameter, µm)	18.6	65.9	29.1	27.8	8.7

Legend: Min, minimum; Max, maximum; N, number of samples; SD, standard deviation.

**Table 3:** *H. foliosum* leaf: LM microscopic characteristics.

Statistical Parameters N = 20		Min	Max	Mean	Median	±SD
**Leaf Surface**						
Adaxial epidermal cells	Area (µm^2^)	79.3	802.3	317.4	369.6	168.8
	Number	29.0	59.0	43.5	44.5	7.2
Abaxial epidermal cells	Area (µm^2^)	187.1	650.9	382.4	356.2	154.4
	Number	26.0	100.0	59.9	58.0	17.9
Abaxial stomatal index (SI) (%)		5.1	18.8	11.5	11.9	3.6
**Transverse Section**						
Mesophyll thickness (µm)		94.3	177.9	126.9	124.3	18.9
Adaxial cuticle thickness (µm)		1.7	6.9	4.0	3.9	1.7
Abaxial cuticle thickness (µm)		1.5	6.8	3.6	3.2	1.4
Palisade parenchyma length (µm)		28.6	49.1	36.6	36.2	5.1
Spongy parenchyma length (µm)		56.9	144.7	89.1	87.7	17.8
Palisade parenchyma/spongy parenchyma ratio		0.2	0.6	0.4	0.4	0.1
Translucent gland diameter (µm)		18.1	66.4	44.7	43.1	11.2
Midrib thickness (µm)		294.0	882.1	719.3	754.9	148.1
Phloem thickness (µm)		40.5	92.5	63.8	62.3	15.6
Xylem thickness (µm)		65.4	97.3	80.2	77.7	8.8
Secretory canals—type B diameter (µm)		11.2	59.7	32.2	27.7	12.2

Min, minimum; Max, maximum; N, number of samples; SD, standard deviation.

**Table 4:** *Hypericum foliosum* extracts’ antioxidant activity.

	DPPH	FRAP	PA
	IC_50_ (µg/mL) ±SD	mg AAE/g dE ± SD	IC_50_ (µg/mL) ±SD
**Hf. E**	490.5 ± 20.6	351.9 ± 12.15	180.9 ± 6.2
**Hf. DM**	695.7 ± 29.0	349.5 ± 4.47	154.7 ± 5.3
**Hf. W**	902.2 ± 27.7	346.5 ± 3.31	411.4 ± 14.3
**Ascorbic Acid**	84.4 ± 0.7	/	49.1 ± 0.4

DPPH, 2,2-diphenyl-1-picrylhydrazyl assay; FRAP, ferric reducing antioxidant power assay; PA, phosphomolybdic acid assay; Hf.E, hydroethanolic extract; Hf.DM, dichloromethane/methanol extract; Hf.W, water extract.

**Table 5:** *Hypericum foliosum* extracts’ selective cytotoxic activity.

	MDA-Mb-436	A549	HCT8
	IC_50_ (µg/mL) ± SD	IC_50_ (µg/mL) ± SD	IC_50_ (µg/mL) ± SD
**Hf. E**	108.6 ± 11.9	76.3 ± 9.1	119.9 V 10.5
**Hf. DM**	71.5 ± 4.0	27.3 ± 2.1	37.7 ± 5.5
**Hf.W**	> 200	>200	>200

MDA-Mb-436, breast cancer cells; A549, lung carcinoma epithelial cells; HCT8, human colon cancer cells; Hf.E, hydroethanolic extract; Hf.DM, dichloromethane/methanol extract; Hf.W, water extract.

**Table 6:** LC-UV/DAD gradient program conditions.

Time (Min)	%H_2_O + 0.05%TFA	%MeCN	%MeOH
0	98	2	0
12	85	15	0
25	70	20	0
35	40	50	10
40	31	54	10
49	21	64	15
55	15	70	15
80	5	80	15
81	98	2	0
90	90	2	0

## Data Availability

Not Applicable.

## References

[1] Ramalhete N., Machado A., Serrano R., Gomes E.T., Mota-Filipe H., Silva O. (2016). Comparative study on the in vivo antidepressant activities of the Portuguese Hypericum foliosum, Hypericum androsaemum and Hypericum perforatum medicinal plants. Ind. Crops Prod..

[2] Santos P.A., Figueiredo A.C., Barroso J.G., Pedro L.G., Scheffer J.J. (1999). Composition of the essential oil of Hypericum foliosum Aiton from five Azorean islands. Flavour Fragr. J..

[3] Ruhfel B.R., Bittrich V., Bove C.P., Gustafsson M.H., Philbrick C.T., Rutishauser R., Xi Z., Davis C.C. (2011). Phylogeny of the clusioid clade (*Malpighiales*): Evidence from the plastid and mitochondrial genomes. Am. J. Bot..

[4] Nürk N.M., Crockett S.L. (2011). Morphological and Phytochemical Diversity among *Hypericum* Species of the Mediterranean Basin. Med. Aromat. Plant Sci. Biotechnol..

[5] do Amaral Franco J. (1971). Nova Flora de Portugal.

[6] Robson S.L.C.N.K.B. (2012). Taxonomy and Chemotaxonomy of the Genus Hypericum. Med. Aromat. Plant Sci. Biotechnol..

[7] Rainha N., Lima E., Baptista J. (2011). Comparison of the endemic Azorean Hypericum foliosum with other Hypericum species: Antioxidant activity and phenolic profile. Nat. Prod. Res..

[8] EMA (2009). Community Herbal Monograph Hypericum perforatum L., Herba (Well-Established Medicinal Use).

[9] Cardile A., Zanre V., Campagnari R., Asson F., Addo S.S., Orlandi E., Menegazzi M. (2023). Hyperforin Elicits Cytostatic/Cytotoxic Activity in Human Melanoma Cell Lines, Inhibiting Pro-Survival NF-kappaB, STAT3, AP1 Transcription Factors and the Expression of Functional Proteins Involved in Mitochondrial and Cytosolic Metabolism. Int. J. Mol. Sci..

[10] Deng X., Xia J., Hu B., Hou X.C., Pu X.D., Wu L. (2022). Hyjapones A-D, trimethylated acyphloroglucinol meroterpenoids from Hypericum japonicum thunb. With anti-inflammatory activity. Phytochemistry.

[11] Zhai X., Chen Y., Han X., Zhu Y., Li X., Zhang Y., Lu Y. (2022). The protective effect of hypericin on postpartum depression rat model by inhibiting the NLRP3 inflammasome activation and regulating glucocorticoid metabolism. Int. Immunopharmacol..

[12] Rafailovska E., Tushevski O., Shijakova K., Simic S.G., Kjovkarovska S.D., Miova B. (2023). Hypericum perforatum L. extract exerts insulinotropic effects and inhibits gluconeogenesis in diabetic rats by regulating AMPK expression and PKCepsilon concentration. J. Ethnopharmacol..

[13] Rainha N., Lima E., Baptista J., Rodrigues C. (2011). Antioxidant properties, total phenolic, total carotenoid and chlorophyll content of anatomical parts of *Hypericum foliosum*. J. Med. Plants Res..

[14] Arruda M., Rainha N., Barreto M., Lima E., Baptista J. (2010). Acetylcholinesterase inhibition properties of *Hypericum foliosum* Aiton. Planta Med..

[15] Gibbons S., Moser E., Hausmann S., Stavri M., Smith E., Clennett C. (2005). An anti-staphylococcal acylphloroglucinol from Hypericum foliosum. Phytochemistry.

[16] Moura M. (1998). Conservation ofHypericum foliosum aiton, an endemic azorean species, by micropropagation. Vitr. Cell. Dev. Biol..

[17] Caldeira G.I., Gouveia L.P., Serrano R., Silva O.D. (2022). Hypericum Genus as a Natural Source for Biologically Active Compounds. Plants.

[18] Bottega S., Garbar F., Pagni A.M. (1999). Secretory Structures in Hypericum elodes L. (Hypericaceae). I. Preliminary Observations.

[19] Perrone R., De Rosa P., De Castro O., Colombo P. (2013). A further analysis of secretory structures of some taxa belonging to the genus Hypericum (Clusiaceae) in relation to the leaf vascular pattern. Turk. J. Bot..

[20] Machado A.M.P. (2012). Contribuição para o Conhecimento do Perfil Metabolómico e das Potencialidades Farmacológicas de *Hypericum foliosum* Aiton. Master’s Thesis.

[21] Serrano R., Ferreira P., Gomes E.T., Silva O. (2008). The Use of SEM and Light Microscopy for the Characterization of Hypericum foliosum Aerial Part as a Medicinal Plant. Microsc. Microanal..

[22] Tekin M. (2017). Pharmacobotanical study of Hypericum thymopsis. Rev. Bras. Farmacogn..

[23] Fornasiero R.B., Bianchi A., Pinetti A. (1998). Anatomical and Ultrastuctural Observations inHypericum perforatumL. Leaves. J. Herbs Spices Med. Plants.

[24] Ciccarelli D., Andreucci A.C., Pagni A.M. (2001). The “black nodules” of Hypericum perforatum L. subsp. perforatum: Morphological,anatomical, and histochemical studies during the course of ontogenesis. Isr. J. Plant Sci..

[25] Ciccarelli D. (2001). Translucent Glands and Secretory Canals in Hypericum perforatum L. (Hypericaceae): Morphological, Anatomical and Histochemical Studies During the Course of Ontogenesis. Ann. Bot..

[26] Perrone R., Rosa P., Castro O., Colombo P. (2013). Leaf and stem anatomy in eight Hypericum species (Clusiaceae). Acta Bot. Croat..

[27] Osinska B.L.E. (2010). Shoot anatomy and secretory structures in *Hypericum* species (*Hypericaceae*). Bot. J. Linn. Soc..

